# Diminished gray matter density mediates chemotherapy dosage-related cognitive impairment in breast cancer patients

**DOI:** 10.1038/s41598-018-32257-w

**Published:** 2018-09-14

**Authors:** Xiu Li, Haijun Chen, Yue Lv, Herta H. Chao, Liang Gong, Chiang-Shan R. Li, Huaidong Cheng

**Affiliations:** 1 Department of Oncology, the Second Affiliated Hospital of Anhui Medical University, Hefei, Anhui 230601 China; 2 Department of Oncology, People’s Hospital of Yuxi City, the Sixth Affiliated Hospital of Kunming Medical University, Yuxi, Yunnan 653100 China; 3 Cancer Center, VA Connecticut Healthcare System, West Haven, CT 06516 USA; 4 Department of Internal Medicine, Yale University School of Medicine, New Haven, CT 06520 USA; 5 Department of Neurology, Affiliated ZhongDa Hospital, School of Medicine, Southeast University, Nanjing, Jiangsu 210009 China; 6 Department of Psychiatry, Yale University School of Medicine, New Haven, CT 06520 USA

## Abstract

To investigate chemotherapy dosage-related cognitive impairment and its neural mechanisms in breast cancer (BC) patients. Twenty-eight breast cancer patients after each chemotherapy cycle and matched 29 healthy control subjects underwent structural magnetic resonance imaging. Voxel-based morphometry analysis was performed to compare group differences in the gray matter for the whole brain. Furthermore, mediation analysis was conducted to explore the role of brain structures in chemotherapy dosage-related cognitive impairment. Voxel-based morphometry analysis was performed in gray matter for the whole brain of BC patients after chemotherapy. The results revealed that the gray matter density in the left inferior frontal gyrus, right middle frontal gyrus, right fusiform area, and bilateral cerebellum was decreased in the BC patients compared to controls. The number of chemotherapy cycles was negatively associated with general cognitive capacity, verbal fluency and digit span performance in the BC patients. In addition, decreased gray matter density in the right middle frontal gyrus could mediate the chemotherapy dosage effects on verbal fluency performance. These findings indicate that the dose-response relationship between chemotherapy and cognitive impairment may depend on the decreases in gray matter density of the frontal cortical structures.

## Introduction

Chemotherapy-related cognitive impairment has been reported in many studies of breast cancer patients (for a review^1^). Executive function, processing speed, attention, and memory are frequently identified as cognitive functions vulnerable to chemotherapy in this patient population^2–4^. Both cross-sectional and prospective longitudinal neuropsychological studies demonstrated the greatest influence of chemotherapy on cognitive function up to six months posttreatment^5^, with partial recovery in the subsequent years^6^. Although multiple factors, including age, fatigue and stress may impact cognitive function, cumulative chemotherapy dosage appears to play an outsized role in determining chemotherapy-related cognitive decline^7^. For instance, Collins and colleagues found that breast cancer patients undergoing chemotherapy showed a progressive decline over the course of treatment in overall cognitive capacity, as well as in working memory and processing speed, supporting the dosage effects of chemotherapy on cognitive functions^8^. However, the neural mechanisms underlying these dosage effects have not been investigated.

Numerous neuroimaging studies have reported alterations in brain structure and function following chemotherapy in cancer patients^9^. Both structural and functional brain changes have been found to be related to chemotherapy-related cognitive impairment in breast cancer patients^10–12^. On the other hand, whereas cerebral GM volumes were consistently decreased, functional imaging studies have reported both increased and reduced activations following chemotherapy^9^. In breast cancer patients, the structural cerebral changes were noted in both gray matter (GM) and white matter (WM)^13^. The GM volumes were decreased in the prefrontal cortex, precuneus, temporal cortex, hippocampus and cerebellum, and the fractional anisotropy, an index of the integrity of WM tracks, was decreased in the frontal, parietal and occipital tracts after chemotherapy^14–16^. However, whether and how chemotherapy dosage, which is an important risk factor for chemotherapy-related cognitive impairment, influences the brain has not been investigated.

In the present study, we aimed to investigate the chemotherapy dosage-related cognitive impairment and mediating roles of cerebral structural changes in this relationship in breast cancer patients. First, with voxel-based morphometry, we examined GM volume differences between 28 breast cancer patients undergoing chemotherapy (CC group) and 29 matched control subjects. Second, we explored the relationship between the number of chemotherapy cycles and cognitive impairment in the CC group. Third, we conducted a mediation analysis to investigate whether the altered brain structures mediated the relationship between chemotherapy dosage and cognitive impairment in the patients. We hypothesized that the GM volumes in the frontal and temporal cortex and the cerebellum were decreased in the CC group and that altered GM volumes mediated the dosage-related cognitive impairment in the patients.

## Results

### Demographic information and neuropsychological data

As illustrated in Table 1, there were no significant differences on demographics or in whole GM density between the two groups (*p’s* > 0.05). Compared with the CN group, the CC group showed a significantly worse cognitive function as assessed by MMSE (*t* = 8.63, *p* < 0.001), DS (*t* = 2.51, *p* = 0.015), and VFT score (t = 5.04, *p* < 0.01).

**Table 1:** Demographic and neuropsychological data for all participants.

	CC (n = 28)		CN (n = 29)		*T*	*p* value
	Mean	SD	Mean	SD		
Age	49.21	8.15	49.27	8.59	0.03	0.978
Education (years)	10.15	2.17	10.79	2.96	0.79	0.434
GM (ml)	603.65	44.26	617.16	61.61	0.94	0.47
MMSE	25.71	1.94	29.17	0.92	8.63	<0.001
DS	5.53	1.40	6.34	1.01	2.51	0.015
VFT	8.75	2.46	11.79	2.09	5.04	<0.001
Number of chemotherapy cycles (4–13)	6.00	2.03	—	—	—	—

Notes: Abbreviations: SD, standard deviation; CN, cognitively normal; CC, breast cancer patients after chemotherapy; GM, gray matter; MMSE, Mini-Mental State Examination; DS, digit span; VFT, verbal fluency test.

### Group comparison of GM density

**Figure 1:**
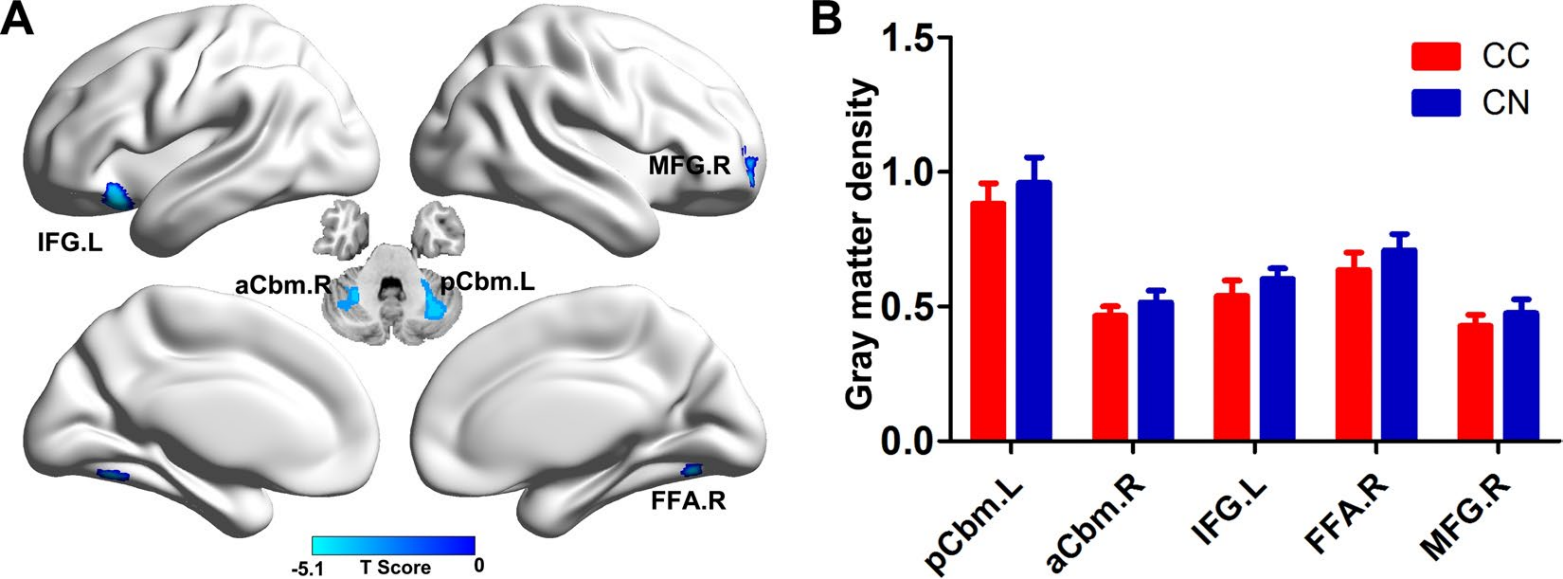
Group difference of gray matter density between CC and CN groups (3dClustSim correction, p < 0.001). (**A**) Brain regions showing gray matter atrophy in CC patients compared to CN subjects; (**B**) Numerical representation of significant difference in gray matter. Abbreviations: CC, breast cancer patients after chemotherapy; CN, cognitively normal subject; pCbm. L, left posterior cerebellum lobe; aCbm. R, right anterior cerebellum lobe; IFG.L, left interior frontal gryus; FFA.R, right fusiform area; MGF.R, right middle frontal gyrus.

As shown in Fig. 1 and Table 2, compared with the CN group, the CC patients showed decreased GM density in several brain regions, including the left posterior cerebellum lobe (pCbm, CC, 0.88 ± 0.07; CN, 0.95 ± 0.09), left interior frontal gyrus (IFG, CC, 0.54 ± 0.06; CN, 0.61 ± 0.03), right anterior cerebellum (aCbm, CC, 0.46 ± 0.04; CN, 052 ± 0.04), right fusiform area (FFA, CC, 0.63 ± 0.06; CN, 0.70 ± 0.06) and right middle frontal gyrus (MFG, CC, 0.43 ± 0.04; CC, 0.48 ± 0.05).

**Table 2:** Brain regions showing gray matter atrophy in the CC groups.

Brain region	BA	Cluster size (mm^3^)	MNI Coordinate (RAI)			Peak *T* score
			X	Y	Z	
pCbm.L	—	6949	−27	−58.5	−27	−4.88
aCbm.R	—	5280	30	−52.5	−34.5	−5.06
IFG.L	47	3480	−33	21	−19.5	−5.04
FFA.R	19	744	28.5	−67.5	−12	−4.34
MFG.R	10	992	30	60	6	−4.21

Abbreviations: CC, breast cancer patients after chemotherapy; MNI, Montreal Neurological Institute space; BA, Brodmann area; pCbm.L, left posterior cerebellum lobe; aCbm.R, right anterior cerebellum lobe; IFG.L, left interior frontal gryus; FFA.R, right fusiform area; MGF.R, right middle frontal gyrus.

### Relationship between cognitive function and number of chemotherapy cycles in CC patients

**Figure 2:**
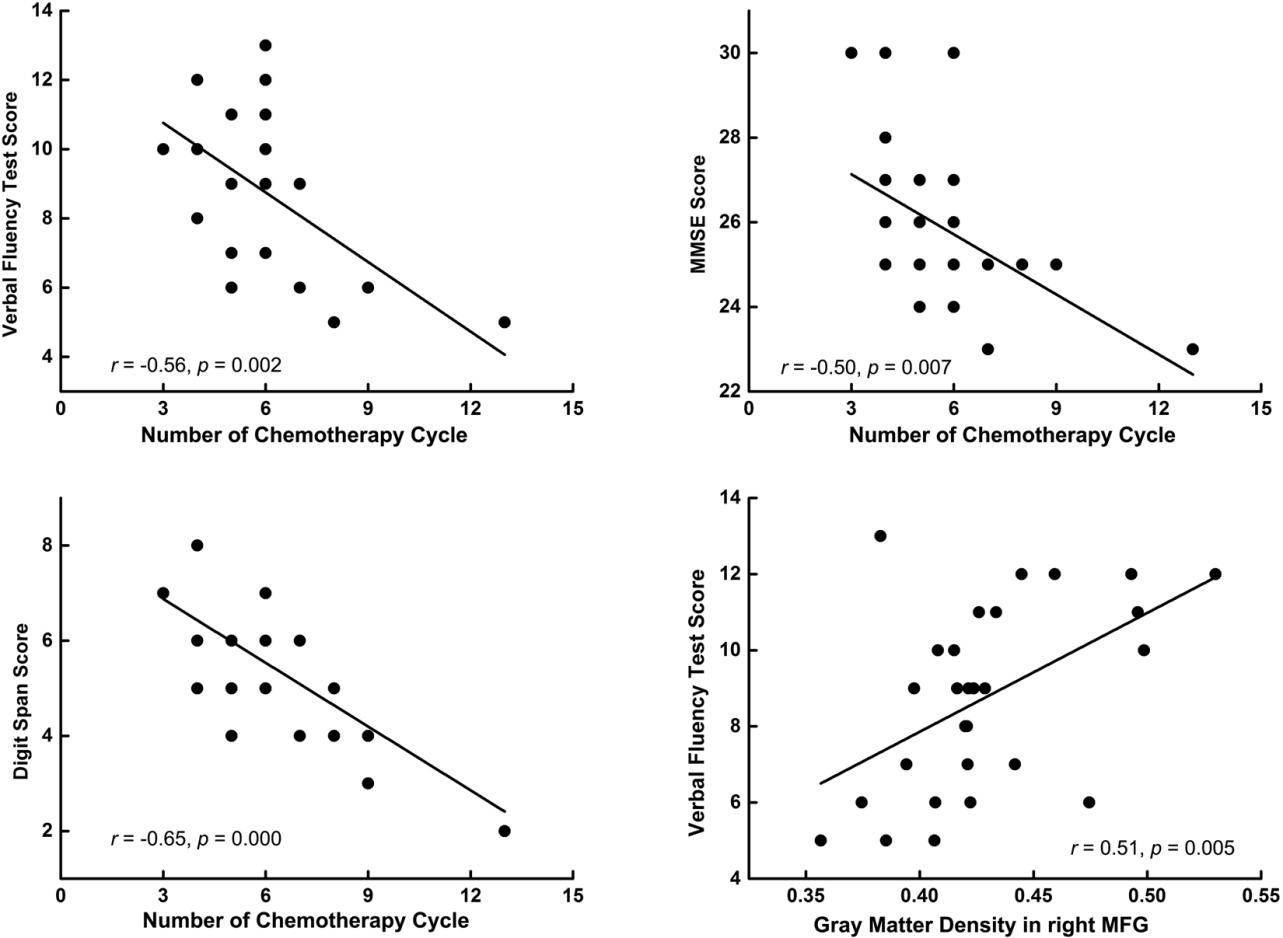
Pearson correlation analyses results. The number of chemotherapy cycles are negatively correlated with cognitive function in CC patients, while the gray matter density in the right MFG is positively correlated with the VFT score in CC patients. Abbreviations: MMSE, Mini-Mental State Examination; DS, digit span; VFT, verbal fluency test; MFG, middle frontal gyrus; CC, breast cancer patients after chemotherapy.

As illustrated in Fig. 2, the number of chemotherapy cycles was significantly and negatively correlated with the MMSE score (*r* = −0.50, *p* = 0.007), DS score (*r* = −0.65, *p* < 0.001), and VFT score (*r* = −0.56, *p* = 0.002) in the CC group. These correlations remained significant after correction for multiple comparisons.

### Cognitive significance of altered GM density in CC patients

The Pearson correlation analyses revealed that the GM density in the right MGF was positively correlated with VFT scores in CC group (*r* = 0.51, *p* = 0.005) (Fig. 2). No other correlations between GM density and cognitive measures were significant.

### Mediation analysis

**Figure 3:**
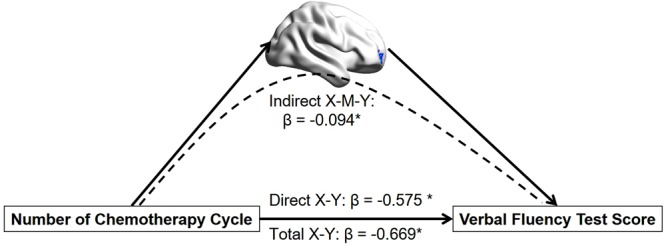
The mediation analysis result. It shows that the GM density of the right MFG mediated the impact of the number of chemotherapy cycles on VFT performance in CC patients. Abbreviations: MFG, middle frontal gyrus; VFT, verbal fluency test; CC, breast cancer patients after chemotherapy.

The mediation analysis revealed that there was a significant indirect effect of the GM density of the right MFG on the relationship between the number of chemotherapy cycles and the VFT performance in the patients. As shown in Fig. 3, the GM density of the right MFG negatively mediated the effects of the number of chemotherapy cycle on VFT score in the patients (*indirect effect*, *β* = −0.09, 95% *CI* = [−0.373, −0.061]). That is, more chemotherapy cycles with a higher atrophy in right MFG predicted a lower VFT score in CC patients.

## Discussion

In the present study, we investigated the chemotherapy dosage effect on cognitive impairment in the breast cancer patients and whether and how the brain structural changes could influence dosage-related cognitive impairment. There were three main findings: (1) breast cancer patients undergoing chemotherapy demonstrated cognitive impairment, and the number of chemotherapy cycles was negatively correlated with cognitive performance in the patients; (2) breast cancer patients showed decreased gray matter density in the frontal gyrus, fusiform area and cerebellum; (3) the decreased gray matter density in the right MFG mediated the chemotherapy dosage-related impairment in executive function in breast cancer patients. These findings provided structural neural evidence for executive dysfunction in breast cancer patients receiving chemotherapy treatment.

First, we replicated chemotherapy-related cognitive impairment in breast cancer patients, as shown earlier for general cognitive ability, working memory and executive function in other investigators’ and our previous studies^17^. However, unlike most of the previous studies that examined cognitive performance one month or longer after chemotherapy, all subjects in the present study were studied as they were receiving chemotherapy. Thus, with this experimental design, we were able to evaluate a dose-response relationship between chemotherapy and cognitive impairment^18^. Consistent with previous studies, we showed that general cognitive capacity, working memory, and executive function were linearly correlated with the number of chemotherapy cycles, in support of a dosage effect of chemotherapy on cognitive impairment in breast cancer patients^8,9^.

Second, we demonstrated that the brain structural changes may occur after just a few cycles of chemotherapy. Brain regions including the frontal gyrus, fusiform area and cerebellum showed decreases in gray matter density in the patients after chemotherapy. These structural brain alterations have been reported previously^19^. For example, McDonald and colleagues found that the GM density was not significantly changed in breast cancer patients before chemotherapy but showed decreased GM density in bilateral MFG and cerebellum after 1 month of chemotherapy^20^. We also found that decreased gray matter density in the right MFG was correlated with impaired verbal fluency performance in the breast cancer group. Verbal fluency tests involve multidimensional cognitive processes such as attention, short-term memory, mental flexibility, speed processing, and long-term vocabulary storage^21^. Verbal fluency has been viewed as a critical component of executive function and engages the prefrontal cortex^12,22^. Previous studies have also reported that a reduced frontal gray matter density is associated with self-reported executive dysfunction^23^. The current findings thus extend our understanding of the cognitive side effects of chemotherapy by elucidating the time window of the effects of chemotherapy on brain and cognition.

Third, although we did not find a direct association between the number of chemotherapy cycles and altered GM density, the mediation analysis revealed that the altered GM density in the right MFG may mediate the chemotherapy dosage-related executive dysfunction in breast cancer patients. The MFG (Brodmman area 46) is critical to executive function and semantic processing^24^. A recent meta-analysis of neuroimaging studies suggested that the anterior part of the middle frontal gyrus plays a central role in language production, a process central to performance in verbal fluencies tests^25^. The latter finding along with the current results supported the mediator role of GM density of MFG in the chemotherapy dose-related verbal fluency impairment in breast cancer patients. The current results also suggest verbal fluency and, more broadly, right frontal cortical functioning as a potential therapeutic target in the cognitive rehabilitation in cancer patients undergoing chemotherapy treatment.

### Clinical Implications

Chemotherapy-related cognitive impairment has been known for several decades, but there are no proven interventions that may ameliorate these side effects. In a recent case study, Knotkova and colleagues reported the effects of transcranial direct current stimulation (tDCS) of the dorsolateral prefrontal cortex in improving executive functions in patients receiving chemotherapy^26^. By precisely locating the brain regions impacted by chemotherapy, the current and future studies may provide potentially more precise targets for brain stimulation interventions.

### Limitations

The present study has several limitations. First, most of the patients recruited in the study are still undergoing chemotherapy, so the chemotherapy effects on cognition and brain should be considered “acute” and findings should be qualified with this consideration. More studies are needed to evaluate how these cognitive side effects may recover over time^13^. In particular, longitudinal follow-up would allow investigators to confirm the mediating roles of brain structural alterations on longer-term cognitive impairment as a result of chemotherapy. Second, this study involved a relatively small sample, and previous studies have reported brain structural alterations before adjuvant treatment^27^. Thus, further studies should include prechemotherapy scans as a control to confirm and extend the present findings. Third, as a preliminary study, the neuropsychological tests used in this study were limited (just MMSE screening, DS and VFT), and future studies should use a more comprehensive cognitive evaluation method to determine which cognitive domain is the most significantly influenced by chemotherapy. Fourth, the chemotherapy regimens were different across patients. Recent research has suggested different neurotoxic profiles of anthracycline and non-anthracycline-based chemotherapy in breast cancer survivors^28^. Future studies with a larger sample would be needed to examine the potentially different neural and cognitive side effects of different chemotherapy regimens.

## Conclusion

We showed chemotherapy dose-response cognitive impairment in breast cancer patients. Chemotherapy may lead to decreases in gray matter density in the frontal cortex and cerebellum during chemotherapy in breast cancer patients. Importantly, the gray matter density of the right middle frontal gyrus could mediate the chemotherapy dosage-related executive dysfunction in breast cancer patients. These findings support a dosage effect of chemotherapy on cognitive impairment and highlight a potential neural basis of these side effects. These brain regions may serve as a target in cognitive rehabilitation of chemotherapy-related cognitive impairment in cancer patients.

## Methods and Materials

### Participants

Twenty-eight breast cancer patients who underwent postoperative adjuvant chemotherapy (CC group) were recruited from the Second Affiliated Hospital of Anhui Medical University. Twenty-nine age and education-matched cognitively normal (CN) female subjects were recruited from Jan 1, 2016, to Dec 31, 2017. Because a previous study reported that the regional brain structural changes might partial recover over time^29^, all subjects in the present study were recruited during one week after each chemotherapy cycle. All subjects were right-handed. The study was approved by the Research Ethics Committee of the Affiliated Second Hospital of Anhui Medical University, and all subjects provided their informed consent. All research was performed in accordance with relevant guidelines and regulations.

Patients in the CC group were recruited according to the following criteria: (1) de novo breast cancer confirmed by postoperative pathology; (2) standard-dose chemotherapy treatment with doxorubicin (175 mg/m^2^) and paclitaxel (40 mg/m^2^) for each chemotherapy cycle, but without hormonal treatment; (3) normal cognitive function with the Mini-Mental State Examination (MMSE) score of ≥24; (4) normal daily activities with the Karnofsky Performance Scale(KPS) score of ≥80; (5) no impairment in vision, hearing or language. Breast cancer patients with the following conditions were excluded: (1) cachexia and distant metastasis; (2) treatment with hormone or radiation therapy; (3) psychiatric symptoms such as anxiety and depression according to the evaluation of the Hamilton Rating Scale for Depression (HAMD) and Hamilton Rating Scale for Anxiety (HAMA), since psychological distress may be associated with poor cognitive function^30^; (4) any neurological or psychiatric illnesses that may lead to cognitive dysfunction; (5) a history of alcohol/drug dependence; (6) severe diseases of the heart, liver, kidney, brain, and hematopoietic system; (7) contraindication for MRI. The control participants were recruited according to the following criteria: (1) no complaint of memory loss, no severe medical diseases or any neurological or psychiatric illnesses including alcohol and substance use disorders; (2) MMSE score of ≥24; (3) normal brain CT and MRI.

### Neuropsychological background tests

A battery of neuropsychological tests was administered to all subjects to assess general cognitive function, working memory and executive functions. MMSE was administered to assess the general cognitive functions. The Verbal Fluency Test (VFT) was used to evaluate the executive function where the subjects were asked to name all of the animals they could in one minute. Digit Span (DS) was used to measure working memory in which subjects were asked to recall a series of numbers after hearing them in randomized order. The total score was determined by the number of digits recalled in the correct serial order.

### Image acquisition

MR images were acquired using a Siemens Verio 3.0-T scanner (Siemens, Erlangen, Germany) with a 12-channel head coil. The subjects were instructed to relax and close their eyes and not to think of specific things during the scan. Their ears were occluded with earplugs. A pair of stabilizers immobilized their heads. All participants reported that they complied with these instructions. For structural image scanning, a 3D-magnetization prepared rapid gradient-echo sequence was used with TR = 1900 ms; TE = 2.48 ms; FA = 9°; acquisition matrix = 256 × 256; FOV = 250 × 250 mm; thickness = 1.0 mm; gap = 0 mm, number of slices = 176. Additionally, routine axial T2-weighted mages were obtained to exclude subjects with major white matter changes, cerebral infarction, or other lesions. Each MR scan lasted for approximately 10 minutes.

### Image processing

The structural image analysis was performed with voxel-based morphometry (VBM). All anatomical data were processed using VBM8 toolbox (http://dbm.neuro.unijena.de/vbm) with the SPM12 software package (http://www.fil.ion.ucl.ac.uk/spm) implemented in MATLAB 8.0 (Mathworks Inc., Sherborn, MA, USA). First, all T1-wighted structural images were displayed and manually reoriented to place the anterior commissure at the origin of the 3D Montreal Neurological Institute (MNI) space in SPM12. Second, segmentation of the brain tissues was conducted using the likelihood of each voxel of the brain images to be classified as GM, white matter (WM), and cerebrospinal fluid (CSF). Third, the DARTEL algorithm was used to create the study group-specific template. The high-resolution structural GM images for all participants were spatially normalized in the same stereotactic space based on the MNI template using 12 affinity transformations and 16 nonlinear iterations. Third, the second segmentation was performed by using spatial normalization parameters for the native images based on a study group-specific GM template, followed by interpolation of voxel size 2 × 2 × 2 mm^3^ using the trilinear interpolation method recommended by SPM8. Fourth, the absolute GM density was modulated for each subject by Jacobian determinants derived from the spatial normalization by DARTEL. Finally, all segmented GM images were smoothed using 8-mm full-width at a half-maximum (FWHM) Gaussian kernel to increase the signal-to-noise ratio. Following the described procedures, statistical analysis was carried out with a voxelwise comparison of the GM density between CC and CN.

### Statistical analysis

All the statistical analyses were conducted by LG, who was blinded to the experimental grouping.

### Demographic and neuropsychological data

Two sample t-tests were employed to compare the demographic data and neuropsychological performances between the two groups. All values are presented as the mean and standard deviation. *p* < 0.05 was considered statistically significant. All statistical analyses were conducted with SPSS 20.0 software (SPSS, Inc., Chicago, IL, USA).

### VBM analysis

The VBM analysis of gray matter density data was performed using the Resting-State fMRI Data Analysis Toolkit (REST) toolbox (http://restfmri.net/forum/index.php). A voxelwise based two-sample T test was conducted to compare GM density between the two groups, with age and years of education as covariates. We applied explicit masking using the population-specific masking toolbox in SPM12 in order to restrict the search within the GM (http://www.cs.ucl.ac.uk/staff/g.ridgway/masking/)^31^. The voxel level significance threshold was set at *p* < 0.005 and was corrected for multiple comparisons at the cluster-level with the latest version of 3dClustSim program in AFNI_16.3.00 (gray matter mask correction (152997 voxels) at the voxel level *p* < 0.001, cluster level *α* < 0.001, κ > 44, and cluster size >352 mm^3^; https://afni.nimh.nih.gov/pub/dist/doc/program_help/3dClustSim.html). GM density was visualized with the BrainNet Viewer (http://www.nitrc.org/projects/bnv/). To illustrate the group difference numerically and to conduct correlation analysis in the CC group, the average GM density of voxels in the brain regions showing group differences was extracted.

### Pearson correlation analysis

Pearson correlation analyses were used to investigate whether the number of chemotherapy cycles influenced the cognitive function of the patients. We used Pearson correlation to explore the association between altered GM density and cognitive impairment in cancer patients. The significance level was set at *p* < 0.05, with correction for multiple comparisons using a Bonferroni adjustment.

### Mediation analysis

We employed mediation analysis to explore the potential relationship among the number of chemotherapy cycles, GM atrophy, and cognitive function. Because of the observed significant correlation between GM density in the right middle frontal gyrus (MFG) and verbal fluency test (VFT) score in the CC group (see Results), we investigated whether the GM density in the right MFG mediated the effect of the number of chemotherapy cycles on VFT performance in CC patients. We used a simple moderation model from PROCESS Marco in SPSS for the mediation analysis (model 4)^32^. This model was described in our previous study^33^. Briefly, this model was based on 10,000 bootstrap samples for the bias corrected bootstrap confidential interval (CI). The indirect effect was determined as significant at a 95% CI that does not include zero, with a null hypothesis that there was no indirect effect. Three-step regression models were constructed, as shown below:

1 $${\rm{Y}}={c}{\rm{X}}+{c}_{{1}}{{\rm{U}}}_{{1}}+{c}_{{2}}{{\rm{U}}}_{{2}}+e{1}$$

2 $${\rm{M}}=a{\rm{X}}+{a}_{{1}}{{\rm{U}}}_{{1}}+{a}_{{2}}{{\rm{U}}}_{{2}}+e{2}$$

3 $${\rm{Y}}=c^{\prime} {\rm{X}}+b{\rm{M}}+{b}_{{1}}{U}_{{1}}+{b}_{{2}}{U}_{{2}}+e{3}$$

where X is the independent variable (number of chemotherapy cycles), Y is the dependent variable (VFT score), M is the mediator (GM density in right MFG), and U_1_ and U_2_ are the years of education and age, respectively. The *direct effect* represents the effect of X on Y independent of the effect of the mediator (M) on Y (path *c’*). The *direct effect* between X and Y is not necessarily a prerequisite for mediation^11^. The *indirect effect*, or the effect of X on Y via M, is estimated as the product of the effect of X on M and the effect of M on Y, controlling for X (*ab* with 95% bootstrap CI). The total effect of X on Y is the sum of the direct and indirect effects (path *c*).
